# Integrating translational bioinformatics into the medical curriculum

**DOI:** 10.5116/ijme.53ae.bc97

**Published:** 2014-06-28

**Authors:** Benedict Yan, Kenneth H.K. Ban, Tin Wee Tan

**Affiliations:** 1 Nuffield Division of Clinical Laboratory Sciences, Radcliffe Department of Medicine, Oxford, UK; 2 Department of Biochemistry, Yong Loo Lin School of Medicine, National University of Singapore, Singapore

With the arrival of genomic medicine, there is a growing consensus for a need to enhance the genomic literacy of healthcare professionals.^1^^-^^3^ The increasing adoption of genomic and other high-throughput ‘omics technologies in clinical labs requires that healthcare professionals, in particular, physicians be equipped with the knowledge and skills to understand and interpret genomic and other high-throughput ‘omics data for patient care. Indeed, this critical need was articulated in a report by the Human Genomics Strategy Group^4^ (established as part of the UK Government's response to the 2009 House of Lords Inquiry into genomic medicine) that recommended "urgent action" be taken by the Department of Health to "ensure that workforce developments do not lag behind service developments, and that an appropriately skilled workforce is available".In this opinion piece, we argue that enhancing the literacy of physicians in the understanding and interpretation of genomics and other ‘omics data should be integrated into the core medical curriculum, and we propose a set of foundational concepts/skills to be taught as a translational bioinformatics module.

## Addressing the challenge of high-throughput genomics and other ‘omics data for physicians

Genome sequencing and other high-throughput 'omics technologies are poised to effect a major paradigm shift in medical care. The increasing accessibility of such technologies to interrogate the genomic, transcriptomic, proteomic, metabolomic, and microbial profile of patients provides a wealth of data that may be used to guide the diagnosis and monitoring of specific diseases, and potentially to infer future health risks.^5^ The potential of ‘omics profiling is exemplified by a recent paper where genomic, transcriptomic, and metabolomics data were used to estimate the disease risk of a healthy individual.^6^ Given the advances in high-throughput technologies, it is anticipated that such profiling would be increasingly available to patients and the general public.This increase in accessibility of ‘omics profiling presents an additional challenge to the physician, as data from ‘omic profiles typically includes representations of complex data and is often difficult to summarize. Physicians therefore need to be conversant with the analysis of such profiling data in order to interpret and translate the data into advice for the patient or into a clinical decision. This need for balanced interpretation of high-throughput data is evident even today; physicians increasingly encounter patients who are familiar with public bioinformatic databases and use commercial genomic services. In these cases, the physician plays a critical role in distilling the patient’s genomic data, and translating them into advice or a clinical decision, which may be in the context of ambulatory or in-patient care. In ambulatory care for example, healthy patients who use genome services would consult a physician to obtain advice on any potential health risks identified by genome sequencing. Here, the physician would need to explain to patients how risk scores are calculated, how the scores should be interpreted and evaluated for their reliability. In an in-patient setting, for instance in oncology, patients who have their tumours sequenced would need the expert opinion of a physician to translate the genomic data into a clinical decision about treatment options.In light of the rapid advances and increasing adoption of ‘omics technologies in the clinic, there is a critical need for physicians to be familiar with the analysis and interpretation of such high-throughput data in order to make clinical decisions and to communicate health risks to patients, who have access to data requiring the expert opinion of physicians. We propose that physicians need to be equipped with a set of skills which fall under the category of 'translational bioinformatics' (considered by some authors as synonymous with clinical bioinformatics^7^), defined by the American Medical Informatics Association (AMIA) as "the development of storage, analytic, and interpretive methods to optimize the transformation of increasingly voluminous biomedical data, and genomic data, into proactive, predictive, preventive, and participatory health”.^8^ The set of interpretive methods in translational bioinformatics is of greatest relevance to physicians, and is based on the understanding of quantitative and statistical methods, familiarity with biomedical databases, and proficiency with bioinformatics tools. These skills of translational bioinformatics will help physicians understand how to evaluate and interpret ‘omics data that we anticipate will be routinely available to both physician and patient.

## A need for incorporating clinical and translational bioinformatics into the core medical curriculum

After more than half a decade of rapid growth, translational bioinformatics is steadily expanding its footprint, from a series of international conferences worldwide driving interest, a slew of journals focusing on various aspects of translational and clinical bioinformatics, and corresponding training and certification programs in leading institutions.^9^ Although garnering interest mostly from non-health professionals to date, there is a general consensus that certain clinical post-graduate specialty disciplines, in particular genetics^10^ and molecular pathology,^11^ require their practitioners to possess some degree of proficiency in bioinformatics. It is our opinion that such bioinformatics training should not be limited to certain post-graduate disciplines, but extended to post-graduate continuing medical education, and to the core medical curriculum.It is anticipated that every future physician, whether in public health^12^ or surgery,^13^ will encounter genomic (or other ‘omic) results in some form that requires the ability to understand how these results were generated, and to interpret the significance of such findings. In a recent JAMA article exploring the clinical implications of whole-genome sequencing in healthy adults, primary care physicians were presented with genomic results in an attempt to understand the burden and cost of clinical follow-up following the identification of pathogenic variants.^14^^, ^^15^

## Proposal for a clinical/translational bioinformatics curriculum

In response to these challenges, we propose that translational bioinformatics be included in the core medical curriculum so that every physician will be equipped with core concepts and skills needed to understand and interpret genomic and ‘omics data, based on a firm grasp of relevant concepts in mathematics, probability, statistics, and computing. The specific concepts and skill sets to be taught in a medical undergraduate clinical/translational bioinformatics module include:

1. Understanding of molecular biology and genetics, with a focus on diseases and high throughput ‘omics technologies
2. Understanding of biostatistical methods (null hypothesis testing, Bayesian inference) with an emphasis on recent developments in analyzing high-throughput data.
3. Familiarity with biomedical databases, the sources of their data, their accuracy, reliability and quality of curation, particularly those readily accessible to patients.
4. Proficiency with bioinformatics tools for analysis, interrogation and visualization of data (for example, the Integrative Genomics Viewer^16^).
5. Familiarity with genetic counseling, with emphasis on communicating risks and choices.
6. Understanding of ethical and legal issues involving ‘omics data, including privacy, confidentiality, data protection.

How might this module be taught? We believe that didactic lectures, initially focusing on the core concepts, need to be supplemented with practical hands-on sessions that incorporate representative medical case scenarios. For instance, students can be taken through a case of a healthy patient with genomic data that requires the use of bioinformatics tools to interrogate potential genomic variants and assess their significance by cross-referencing them to biomedical databases on gene function. In this way, translational bioinformatics may be reinforced as a practical skill that is needed when managing patients with ‘omics profiles.

## Conclusions

In summary, the advent of genomic medicine presents a new challenge for physicians who need to understand and interpret high-throughput ‘omics data in order to communicate them to patients who share in the decision-making process in planning treatments and making healthcare decisions. Accordingly, there is a critical need to provide teaching and training for healthcare professionals, in particular physicians, and this need has been recognized internationally. Several countries, such as the United States and Canada, have delineated a national vision and strategy for genomic medicine. In the UK, Genomics England, with its 100K Genome Project, has embarked on delivering genomics into mainstream healthcare in the National Health Service.^17^At a time when the public is increasingly looking toward the physician for guidance and advice on ubiquitous biomedical, genetics and genomic data, it is important for those responsible for shaping medical education to adopt a proactive approach in defining and incorporating such curricula at different phases of the physicians' education and training. Given the long lead time in changing the medical curriculum, now is as good a time to begin designing such a course that will give our students a head-start in the future of modern medicine.

### Conflict of Interest

The authors declare that they have no conflict of interest.

## References

[1] Brunham LR Hayden MR. Medicine. Whole-genome sequencing: the new standard of care? Science. 2012;336(6085):1112-1113. 10.1126/science.1220967 22654044

[2] Guttmacher AE Porteous ME McInerney JD. Educating health-care professionals about genetics and genomics. Nat Rev Genet. 2007;8(2):151-157. 10.1038/nrg2007 17230201

[3] Saul RA. Genetic and genomic literacy in pediatric primary care. Pediatrics. 2013;132(Suppl 3):S198-202. 10.1542/peds.2013-1032C 24298127

[4] Schonherr C Ruuth K Kamaraj S Wang CL Yang HL Combaret V Anaplastic Lymphoma Kinase (ALK) regulates initiation of transcription of MYCN in neuroblastoma cells. Oncogene. 2012;31(50):5193-5200. 10.1038/onc.2012.12 22286764

[5] Friend SH Ideker T. Point: are we prepared for the future doctor visit? Nat Biotechnol. 2011;29(3):215-218. 10.1038/nbt.1794 21390021

[6] Chen R Mias GI Li-Pook-Than J Jiang L Lam HY Chen R Personal omics profiling reveals dynamic molecular and medical phenotypes. Cell. 2012;148(6):1293-1307. 10.1016/j.cell.2012.02.009 22424236 PMC3341616

[7] Bellazzi R Masseroli M Murphy S Shabo A Romano P. Clinical Bioinformatics: challenges and opportunities. BMC Bioinformatics. 2012;13 Suppl 14:S1. 10.1186/1471-2105-13-S14-S1 23095472 PMC3439676

[8] Butte AJ. Translational bioinformatics: coming of age. J Am Med Inform Assoc. 2008;15(6):709-714. 10.1197/jamia.M2824 18755990 PMC2585538

[9] Brazas MD Lewitter F Schneider MV van Gelder CW Palagi PM. A quick guide to genomics and bioinformatics training for clinical and public audiences. PLoS Comput Biol. 2014;10(4):e1003510. 10.1371/journal.pcbi.1003510 24722068 PMC3983038

[10] ACMG Board of Directors. Points to consider in the clinical application of genomic sequencing. Genet Med. 2012;14(8):759-761. 10.1038/gim.2012.74 22863877

[11] Salto-Tellez M de Castro DG. Next generation sequencing: a change of paradigm in molecular diagnostic validation. J Pathol. 2014; in press. 10.1002/path.4365 24756835

[12] Centers for Disease Control and Prevention. Priorities for public health genomics 2012-2017. 2011 [Accessed 12 June 2014]; Available from: blogs.cdc.gov/genomics/2011/12/01/beyond-base-pairs-to-bedside/

[13] Hottenrott C. Laparoscopic resections and ENCODE-guided genomics to advance surgery and oncology. Surg Endosc. 2014; 28(7):2244-6. 10.1007/s00464-014-3456-3 24566746

[14] Dewey FE Grove ME Pan C Goldstein BA Bernstein JA Chaib H Clinical interpretation and implications of whole-genome sequencing. JAMA. 2014;311(10):1035-1045. 10.1001/jama.2014.1717 24618965 PMC4119063

[15] Feero WG. Clinical application of whole-genome sequencing: proceed with care. JAMA. 2014;311(10):1017-1019. 10.1001/jama.2014.1718 24618961

[16] Thorvaldsdottir H Robinson JT Mesirov JP. Integrative Genomics Viewer (IGV): high-performance genomics data visualization and exploration. Briefings in bioinformatics. 2013;14(2):178-192. 10.1093/bib/bbs017 22517427 PMC3603213

[17] Wright CF Middleton A Burton H Cunningham F Humphries SE Hurst J Policy challenges of clinical genome sequencing. BMJ. 2013;347:f6845. 10.1136/bmj.f6845 24270507

